# miR-144-5p Enhances the Radiosensitivity of Non-Small-Cell Lung Cancer Cells via Targeting ATF2

**DOI:** 10.1155/2018/5109497

**Published:** 2018-04-12

**Authors:** Lei Song, Liping Peng, Shucheng Hua, Xiaoping Li, Lianjun Ma, Jing Jie, Dong Chen, Ying Wang, Dan Li

**Affiliations:** ^1^Department of Respiratory Medicine, The First Hospital of Jilin University, Changchun, Jilin 130021, China; ^2^Department of Pediatrics, The First Hospital of Jilin University, Changchun, Jilin 130021, China; ^3^Endoscopy Center, The China-Japan Union Hospital of Jilin University, 146 Xiantai Street, Changchun, Jilin 130021, China

## Abstract

MicroRNAs (miRNAs or miRs) regulate gene expression at the posttranscriptional level and are involved in many biological processes such as cell proliferation and migration, stem cell differentiation, inflammation, and apoptosis. In particular, miR-144-3p is downregulated in various cancers, and its overexpression inhibits the proliferation and metastasis of cancer cells. However, the role of miR-144-5p in non-small-cell lung cancer (NSCLC), especially radiosensitivity, is unknown. In this study, we found that miR-144-5p was downregulated in NSCLC clinical specimens as well as NSCLC cell lines exposed to radiation. Enhanced expression of miR-144-5p promoted the radiosensitivity of NSCLC cells* in vitro* and A549 cell mouse xenografts* in vivo*. Furthermore, we identified activating transcription factor 2 (ATF2) as the direct and functional target of miR-144-5p using integrated bioinformatics analysis and a luciferase reporter assay. In addition, restoration of ATF2 expression inhibited miR-144-5p-induced NSCLC cell sensitivity to radiation* in vitro* and* in vivo*. Our findings suggest that deregulation of the miR-144-5p/ATF2 axis plays an important role in NSCLC cell radiosensitivity, thus representing a new potential therapeutic target for NSCLC.

## 1. Introduction

Lung cancer is the leading cause of cancer related death and has become an increasingly serious public health burden worldwide [1]. Small cell lung carcinoma (SCLC) and non-small-cell lung carcinoma (NSCLC) are the two main types of lung cancer in terms of clinical and pathological classification. NSCLC accounts for about 80% of all lung cancers and has three primary subtypes: adenocarcinoma (AC), squamous cell carcinoma (SC), and large cell carcinoma. Radiation therapy, a key therapeutic option for thoracic malignancies including lung cancers [2], damages the DNA of tumor cells to induce cellular death. Radiation therapy has been shown to be synergistic with surgical resection and chemotherapy in lung cancer [3]. However, increasing clinical data demonstrate that lung cancer patients show heterogeneous responses to radiation therapy. Recent studies suggest that gene expression is a significant contributor to the radiosensitivity of lung cancer cells [4, 5]. Therefore, with the advancements of precision medicine and next-generation sequencing, it is important to identify the differentially expressed genes in order to meet the trend of personalized lung cancer treatment.

MicroRNAs (miRNAs or miRs) are a class of short endogenous noncoding RNAs containing 20–22 nucleotides, which play an important role in tumor development and progression, including tumor cell proliferation and metastasis, cancer stem cell differentiation, and cell apoptosis [6]. miRNAs recognize and bind to the 3′-untranslated region (UTR) of the target mRNA, leading to degradation of the mRNA and/or suppression of protein translation [7–9]. In recent years, growing evidence has shown that miRNAs also play a pivotal role in the radiation sensitivity of various types of cancers, including lung cancer. For instance, high expression levels of miR-98-5p, miR-302e, miR-495-3p, and miR-613 are significantly correlated with the radiosensitivity of NSCLC patients [10]. In contrast, silencing miR-21 expression in A549 cells has been reported to significantly sensitize cells to radiation through inhibition of the PI3K/AKT pathway [11]. Additionally, miR-21 upregulation has been found associated with an attenuated radiation efficacy and a shorter median survival time of NSCLC patients, whereas inhibition of miR-21 reversed the radioresistance by stimulation of apoptosis [12]. These findings suggest that miRNAs may be potential targets in the development of novel strategies for lung cancer treatment through enhancing radiosensitivity.

A previous study has indicated that the expression of miR-144-5p, miR-144-3p, miR-142-5p, and miR-19a-3p in whole blood extends the lifespan of rats for 2 weeks after radiation [13]. However, little is known about the role of miR-144-5p in modifying the radiosensitivity of lung cancer cells. Therefore, the aim of this study was to investigate the role of miR-144-5p in the radiation response and the underlying molecular mechanisms of radiotherapy resistance in lung cancer cells in cell culture and a xenograft mouse model. We found that miR-144-5p was downregulated in clinical NSCLC tissues and decreased in NSCLC cells after irradiation (IR). Enforced overexpression of miR-144-5p enhanced the radiosensitivity of A549 and H460 cells* in vitro* and* in vivo*. Moreover, we identified activating transcription factor 2 (ATF2) as the direct target of miR-144-5p, which is involved in the radiosensitivity of NSCLC. Our findings suggest that the miR-144-5p/ATF2 axis may serve as a potential target for the treatment of NSCLC.

## 2. Materials and Methods

### 2.1. Lung Cancer Tissue Specimens

Lung cancer and adjacent normal tissue samples were obtained by surgical dissection from patients with primary lung cancer. These samples were obtained with written informed consent at the Department of Respiratory Medicine of The First Hospital of Jilin University (Changchun, China), following the procedures approved by the hospital's Ethics Review Board.

### 2.2. Cell Culture

The human lung cancer cell lines A549 and H460 were purchased from the American Type Culture Collection (Manassas, VA, USA). The normal human embryonic kidney cell line HEK-293T was obtained from the Shanghai Cell Collection (Shanghai, China). The cells were cultured with Dulbecco's modified Eagle medium containing 10% fetal bovine serum and 4 mM glutamine in an incubator with 37°C and 5% CO_2_.

### 2.3. miRNA Transfection and Lentiviral Particle Transduction

Agomir-144 and agomir negative control (agomir-NC) were purchased from RiboBio Co. (Guangzhou, China). Cells were seeded in six-well plates at a density of 1 × 10^5^/cm^2^, and 200 nM agomir-144 or agomir-NC was transfected using Lipofectamine 2000 (Life Science Technology, USA) for 48 h, following the manufacturer's protocol. For ATF2 overexpression, A549 cells were exposed to ATF2 lentiviral particles (Hanheng, Shanghai, China) in 5 mg/mL Polybrene (Sigma-Aldrich, St. Louis, MO, USA), with mock particles as the negative control. After 12 h, the cells were selected using puromycin (1.5 *μ*g/ml, Santa Cruz Biotechnology, Santa Cruz, CA, USA), according to the manufacturer's protocol.

### 2.4. Radiation Treatment

Exponentially proliferating A549 and H460 cells (1 × 10^5^ cells/mL) in 96- or 6-well plates were irradiated at calibrated radiation dose rates of 3.5 Gy/min. Dosimetry was fully calibrated before commencing the experimental protocol. Standard cell growth conditions were maintained for 24 h before treatment to avoid the effects of diurnal variation.

### 2.5. 3-(4,5-Dimethylthiazol-2-yl)-2,5-diphenyltetrazolium Bromide Solution (MTT) Assay

Forty-eight hours after IR of exponentially growing A549 and H460 cells in 96-well plates, 15 *μ*L of MTT (5 mg/mL) was added to each well, and the cells were incubated for 4 h at 37°C. The medium containing MTT solution was then removed, and 150 *μ*L of dimethyl sulfoxide was added to dissolve the formazan product. Spectrophotometric absorbance at 490 nm was determined using a microplate reader (Bio-Rad, model 550, Philadelphia, PA, USA).

### 2.6. Apoptosis Assay

Forty-eight hours after IR, the cells were harvested and washed with phosphate-buffered saline (PBS). The cell pellets were resuspended in 100 *μ*L of binding buffer, and Annexin V-FITC (Boehringer Mannheim, Mannheim, Germany) was added to a final concentration of 1 mg/mL; the mixture was incubated in the dark at room temperature for 15 min. Then, 10 *μ*L of propidium iodide (10 mg/mL) was added to the samples, and the mixture was incubated in the dark at room temperature for 5 min. The samples were analyzed using a FACScan flow cytometer (Becton Dickinson; San Jose, CA, USA). Each assay was performed in triplicate.

### 2.7. RNA Extraction and Reverse Transcription–Polymerase Chain Reaction (RT-PCR)

Total RNA from cells was exacted using TRIzol (Invitrogen, Carlsbad, CA, USA) and used for cDNA synthesis with EasyScript First-Strand cDNA Synthesis SuperMix (TransGen Biotech, Beijing, China). Quantitative real-time PCR was performed in triplicate using the TransStart™ SYBR Green qPCR Supermix (TransGen Biotech) on a 7300 PCR System (ABI, Carlsbad, CA, USA). The primers for miR-144-5p and the internal reference U6 were purchased from RiboBio Co. The primer sequences for ATF2 and the internal reference GAPDH were as follows:

ATF2-F, 5′-CAATCCACTGCCATGGCCTT-3′; ATF2-R, 5′-TCAGATAAAGCCAAGTCGAATCTGG-C-3′; GAPDH-F, 5′-CGGAGTCAACGGATTTGGTCG-3′; and GAPDH-R, 5′-AGCCTTCTACATGGTGGTGAAGAC-3′. The relative RNA levels were calculated using the 2^−ΔΔCt^ method.

### 2.8. Western Blotting

Total proteins were extracted using RIPA buffer (Pierce, Rockford, IL, USA). The protein concentration was determined using Bradford method (Pierce). Equal amounts of proteins (40 *μ*g) were separated on a 10% sodium dodecyl sulfate-polyacrylamide gel and transferred to Immobilon-P membranes (Millipore, Bedford, MA, USA). After blocking with 5% nonfat milk in PBS-Tween-20 for 1 h, the membranes were incubated at 4°C overnight with anti-ATF2 antibody (1 : 500 dilution, Abcam, Hong Kong, China) or monoclonal anti-GAPDH antibody (1 : 5000, Abcam). After washing with Tris-buffered saline and Tween 20, the membranes were incubated with the appropriate horseradish peroxidase-conjugated secondary antibody (1 : 5000, Abcam) at 37°C for 1 h. The immunohistochemical reaction was detected using an ECL Plus Detection kit (Pierce).

### 2.9. Colony Formation Assay

Cells (400 cells/plate) in 3.5-cm plates were treated with 8 Gy radiation and then incubated for 10 days with replacement of fresh media every 3 days. The number of spheres with a diameter greater than 75 *μ*m was counted.

### 2.10. Animal Experiments

The procedures of the animal experiments were approved by the Committee on the Use and Care of Animals of The First Hospital of Jilin University (Changchun, China). Cells in the logarithmic growth phase were harvested and resuspended in PBS. A total of 2 × 10^6^ cells in 0.2 mL of PBS were injected subcutaneously into the left flank of 4-week-old BALB/c male nude mice. For miRNA* in vivo* delivery, 7 days after cell injection, PBS (20 *μ*L), agomir-144 (1 nmol in 20 *μ*L of PBS for each mouse), or the equivalent amount of agomir-NC (RiboBio) was intratumorally injected into the implanted tumor every 3 days for seven times. Mice were irradiated with 4 Gy once per day for 5 days. Every 3 days after injection, the tumor volumes were measured with vernier calipers and calculated as follows: tumor volume (mm^3^) = maximal length (mm) × [perpendicular width (mm)]^2^/2. On day 28 after injection, mice were sacrificed and the tumors were removed. Each group had at least eight mice.

### 2.11. Luciferase Assay

Potential targets of miR-144-5p were searched with TargetScan (http://www.targetscan.org/), revealing that ATF2 is a target of miR-144-5p. In order to verify ATF2 as a target of miR-144-5p, the luciferase reporter vector pCMV-REPORT-ATF2-3′UTR-wt was constructed by inserting the pCMV-REPORT vector with the DNA sequence of ATF2 3′UTR containing a putative miR-144-5p-binding site (ACUAUAG). The control luciferase vector pCMV-REPORT-ATF2-3′UTR-mut, harboring the mutant miR-144-5p binding site (UGCGCGA), was also constructed. The miR-144-5p mimic and control were transfected into HEK-293T cells, respectively, using Lipofectamine 2000 (Invitrogen), followed by the detection of luciferase activity with a Dual-Luciferase® Reporter Assay kit (Promega, Madison, WI, USA).

### 2.12. Statistical Analysis

At least three parallel experiments were conducted for all assays. The two-tailed Student's* t*-test was performed for statistical analysis using SPSS 15.0. *P* < 0.05 was considered statistically significant.

## 3. Results

### 3.1. The Expression of miR-144-5p in Lung Cancer Tissues and A549 Cells Treated with IR

**Figure 1:**
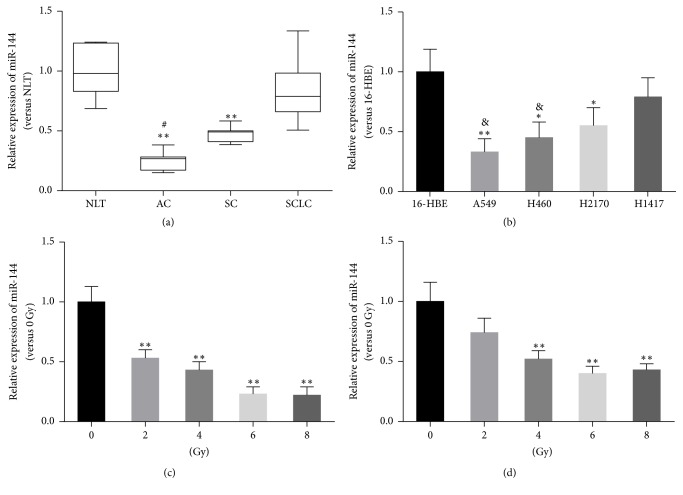
*miR-144-5p expression in lung cancer patient samples and cell lines treated with irradiation.* (a) Relative expression levels of miR-144-5p in normal lung tissue and lung cancer specimens were measured by real-time polymerase chain reaction. NLT, normal lung tissue (*n* = 6); AC, adenocarcinoma (*n* = 12); SC, squamous carcinoma (*n* = 10); SCLC, small cell lung cancer (*n* = 8). ^*∗∗*^*P* < 0.01 versus NLT, ^#^*P* < 0.01 versus SCLC. (b) miR-144-5p expression in the indicated NSCLC cell lines. Data are representative images or expressed as the mean ± standard deviation of each group of cells from three separate experiments. ^*∗*^*P* < 0.05 versus 16-HBE, ^*∗∗*^*P* < 0.01 versus 16-HBE, ^&^*P* < 0.05 versus H1417. (c) miR-144-5p expression in A549 cells and (d) H460 cells after radiation treatment at different doses (0 Gy, 2 Gy, 4 Gy, and 8 Gy). ^*∗∗*^*P* < 0.01 versus 0 Gy.

We first detected the expression of miR-144-5p in lung AC, lung SC, and SCLC. As shown in Figure 1(a), the expression of miR-144-5p in the specimens of AC and SC, but not SCLC, was significantly lower than that of normal lung tissue (NLT). Interestingly, miR-144-5p expression in AC was significantly lower than in SCLC. In addition, miR-144-5p expression was downregulated in NSCLC A549, H460, and H2170 cells, compared to normal human airway epithelial 16-HBE cells; whereas miR-144-5p expression was lower in AC A549 and H460 cells than in SCLC H1417 cells (Figure 1(b)). We further analyzed the relative expression levels of miR-144-5p in A549 and H460 cells treated with IR. IR decreased the expression of miR-144-5p in A549 (Figure 1(c)) as well as in H460 (Figure 1(d)) cells in a dose-dependent manner.

### 3.2. miR-144-5p Enhances IR-Mediated Loss of Cell Viability and Induction of Apoptosis in Lung Cancer Cells

**Figure 2:**
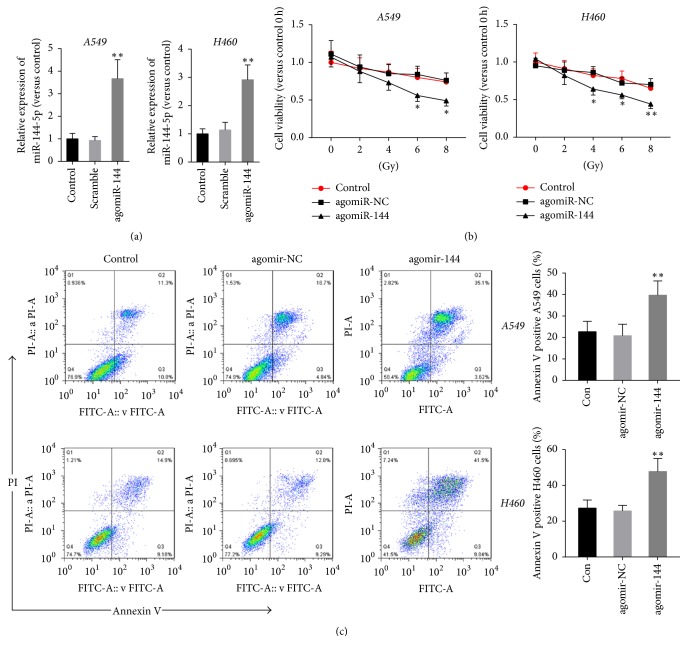
*miR-144-5p regulated A549 and H460 cell viability and apoptosis after irradiation.* (a) The expression of miR-144-5p in control A549 and H460 cells (nontransfected cells), as well as cells transfected with agomir-144 or agomir-NC, was determined using qRT-PCR. (b) Control A549 and H460 cells as well as cells transfected with agomir-144 or agomir-NC were exposed to varying doses of radiation (0, 2, 4, 6, and 8 Gy). MTT assay was used to determine the cell viability 48 h after IR. Cell viability is expressed as the percentage relative to the control at 0 Gy. (c) A549 and H460 cells with or without agomir-144 or agomir-NC transfection were subjected to 8 Gy radiation. Cell apoptosis was assessed by staining with annexin V and propidium iodide 48 h after IR. The percentage of apoptotic cells was determined using flow cytometric analysis. Data are representative images or expressed as the mean ± standard deviation of each group of cells from three separate experiments. ^*∗*^*P* < 0.05 versus agomir-NC. ^*∗∗*^*P* < 0.01 versus agomir-NC.

To explore the role of miR-144-5p in A549 and H460 cells treated with IR, cells were transfected with agomiR-144 or agomir-NC, followed by treatment with different doses of IR. As Figure 2(a) has shown, transfection with agomiR-144 significantly upregulated miR-144-5p expression in A549 and H460 cells compared with those of cells transfected with agomiR-NC, whereas transfection of agomiR-NC has no effects on the expression of miR-144-5p. Cell viability assessment by MTT assay showed that IR decreased the cell viability in a dose-dependent manner; whereas agomir-144, but not agomir-NC, enhanced the loss of cell viability by IR in both A549 and H460 cells (Figure 2(b)). Further apoptosis analysis with annexin V/propidium iodide staining showed that IR at a dose of 8 Gy induced apoptosis in nearly 20% of cells, whereas miR-144-5p significantly enhanced the proapoptotic effects of IR on A549 and H460 cells (Figure 2(c)).

### 3.3. miR-144-5p Enhances IR-Induced Tumor Suppression* In Vitro* and* In Vivo*

**Figure 3:**
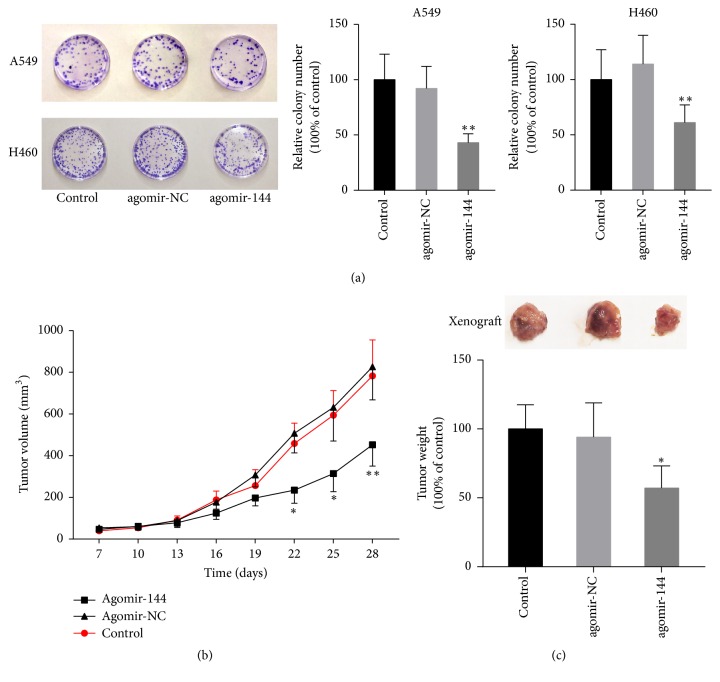
*miR-144-5p enhanced the tumor suppression capability of IR in vitro and in vivo.* (a) A549 or H460 cells transfected with agomir-144 or agomiR-NC and the parental cells (control) were subjected to 8 Gy radiation, followed by a colony formation assay. Colony formation was suppressed in agomiR-144-5p-transfected A549 cells. ^*∗∗*^*P* < 0.01 versus agomir-NC. (b) Seven days after A549 cell injection in mice (six in each group), agomir-144 or agomir-NC was intratumorally injected into the implanted tumor every 3 days for seven times. Mice were irradiated with 4 Gy once per day for the following 5 days. Tumor volumes were measured every 3 days after injection. (c) Tumors were dissected, and the tumor weights were measured on day 28 after inoculation. ^*∗*^*P* < 0.05 versus agomir-NC. ^*∗∗*^*P* < 0.01 versus agomir-NC.

The above results indicated that restoration of miR-144-5p expression enhanced radiation-induced proliferation arrest and apoptosis in lung cancer cells. To confirm this finding, we further investigated the effects of miR-144-5p on radiosensitivity in an* in vitro* colony formation assay and an A549 cell xenograft mouse model. The colony formation assay showed that miR-144-5p overexpression decreased the number of the colonies in A549 and H460 cells treated with IR (Figure 3(a)).* In vivo*, from day 22 after A549 cell subcutaneous injection, mice injected with agomir-144 exhibited a smaller tumor volume, compared to the blank or agomir-NC controls (Figure 3(b)). Furthermore, as shown in Figure 3(c), miR-144-5p overexpression significantly decreased the tumor weights of the xenograft model mice treated with IR.

### 3.4. miR-144-5p Targets ATF2 in Lung Cancer Cells

**Figure 4:**
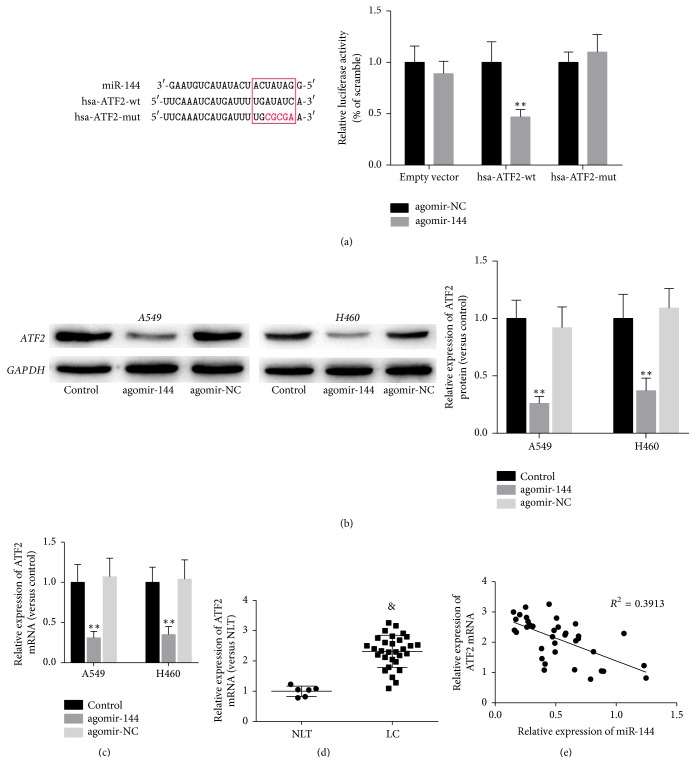
*miR-144-5p targeted ATF2 in lung cancer cells.* (a) The sequence alignment of human miR-144-5p with the 3′UTR of ATF2 is shown. The luciferase reporter constructs hsa-ATF2-wt and hsa-ATF2-mut were made using the seed sequence of miR-144-5p matching the 3′UTR of ATF2 mRNA. HEK293 cells were transiently cotransfected with the indicated plasmids and agomir-144 or agomir-NC for 48 h, and then they were subjected to the luciferase assay. ^*∗∗*^*P* < 0.01 versus agomir-NC. (b, c) A549 and H450 cells were transfected with agomir-144-5p or agomir-NC. The ATF2 protein (b) and mRNA (c) levels were determined by immunoblotting and RT-PCR, respectively. (d) The relative ATF2 mRNA levels in normal lung tissues (NLT, *n* = 6) or lung cancer (LC, *n* = 40) tissues were assessed using quantitative RT-PCR. (e) Correlation between the expression levels of ATF2 and that of mature miR-144-5p in 6 NLT and 40 LC tissues. ^*∗∗*^*P* < 0.01 versus agomir-NC. ^&^*P* < 0.01 versus NLT.

To explore the underlying mechanism by which miR-144-5p enhances the effects of IR in lung cancer cells, we searched for the targets of miR-144-5p with the help of TargetScan, an online database (http://www.targetscan.org/) and found that 2078 transcripts including ATF2 mRNA contain miR-144-5p seed sequences. There is an 8-mer miR-144-5p binding site located in the 3′UTR of ATF2 (Figure 4(a)). For its key role in regulating radiosensitivity of tumor cells [14–16], ATF2 was selected as the potential target of miR-144-5p in present study. To validate this prediction, we performed a luciferase assay using the luciferase expression plasmids containing either wild type or mutant ATF2 3′UTR (hsa-ATF2-wt and hsa-ATF2-mut, respectively). The results demonstrated that miR-144-5p inhibited the luciferase activity in hsa-ATF2-wt but not hsa-ATF2-mut (Figure 4(b)), while agomir-NC had little effect on both plasmids. Next, we treated A549 and H450 cells with agomir-144-5p or agomir-NC and determined ATF2 protein levels by immunoblotting. Agomir-144-5p overexpression decreased ATF2 protein levels in A549 and H450 cells (Figure 4(b)). Consistently, agomir-144-5p treatment resulted in a significant reduction of ATF2 mRNA (Figure 4(c)). Further analysis of the expression of ATF2 mRNA in clinical samples revealed that the level of ATF2 mRNA was lower in normal lung tissues than in lung cancer tissues (Figure 4(d)) and that ATF2 expression was inversely correlated with the miR-144-5p levels (Figure 4(e)).

### 3.5. Restoration of ATF2 Prevents miR-144-5p-Mediated Radiosensitivity of Lung Cancer Cells

**Figure 5:**
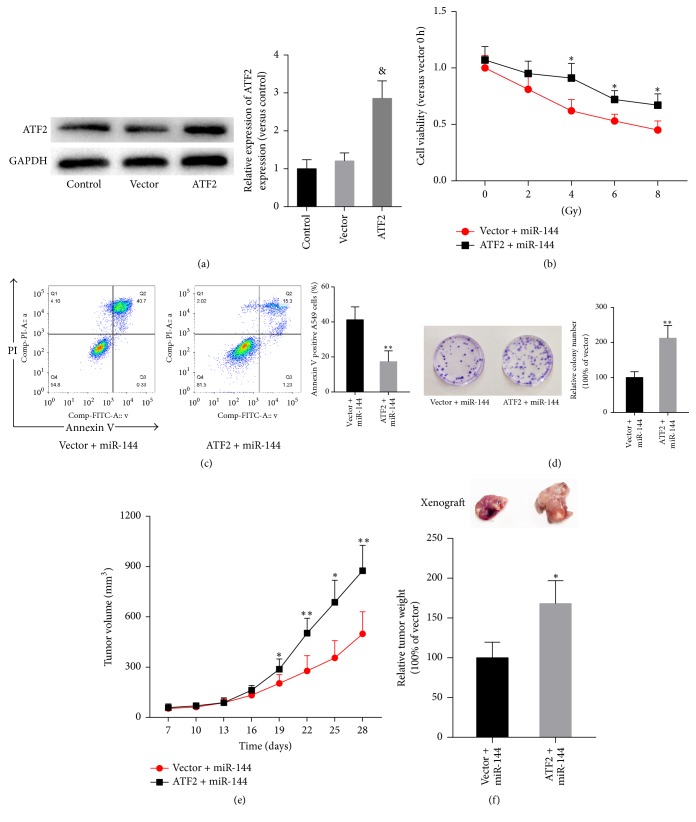
*Restoration of ATF2 expression inhibited miR-144-5p-mediated radiosensitivity of lung cancer cells.* A549 cells with or without ATF2 overexpression were transfected with agomir-144. (a) The levels of miR-144-5p in nontransfected A549 cells (control) and cells transfected with ATF2 lentiviral particles or mock particles (vector) were determined using western blot analysis. (b) After exposure to varying doses of radiation (0, 2, 4, 6, and 8 Gy), MTT assay was used to determine the cell viability. After 8 Gy radiation, cell apoptosis (c) and survival (d) were then determined using Annexin V/propidium iodide staining and the colony formation assay, respectively. (e) A total of 2 × 10^6^ A549 cells stably transfected with ATF2-overexpressing plasmid or vector were injected subcutaneously into mice (6 in each group) to establish a xenograft model. At 7 days after inoculation, agomir-144 was intratumorally injected into the implanted tumor every 3 days for seven times. At the same time, mice were irradiated with 4 Gy once per day for the following 5 days. Tumor volumes were measured every 3 days after injection. (f) Tumors were dissected, and the weights were measured on day 28 after inoculation. Data are representative images or expressed as the mean ± standard deviation of each group of cells from three separate experiments. ^*∗*^*P* < 0.05 versus vector + miR-144. ^*∗∗*^*P* < 0.01 versus vector + miR-144. & represents *P* < 0.01 versus vector.

To assess the role of ATF2 in miR-144-5p-mediated radiosensitivity of lung cancer cells, the radiosensitivity of NSCLC cells after restoration of ATF2 was measured in miR-144-5p-overexpressing cells. As shown in Figure 5(a), ATF2 levels significantly increased in A549 cells transfected with ATF2 lentiviral particles, compared with those of cells transfected with mock particles, which had no effects on ATF2 levels. Ectopic overexpression of ATF2 reversed the viability inhibition (Figure 5(a)) and apoptosis rate (Figure 5(b)) induced by agomir-144 transfection and IR exposure. Similarly, ectopic overexpression of ATF2 abolished the IR-induced loss of the colony formation ability of A549 cells (Figure 5(c)). In addition, restoration of ATF2 significantly inhibited miR-144-mediated suppression of the tumor volume (Figure 5(d)) and weight (Figure 5(e)) in A549 cell xenograft mice exposed to IR.

## 4. Discussion

Radiotherapy is an effective clinical intervention for lung cancer. However, the therapeutic outcome as well as the overall 5-year survival rate depends on the radio susceptibility of the cancer cells. SCLC is the most radiosensitive subtype among the primary lung cancers, while SC exhibits moderate radiosensitivity and AC is resistant to radiotherapy [17, 18]. In addition, it has been reported that NSCLC cell lines are less sensitive to radiation, in contrast to SCLC cell lines [19]. These studies indicate the existence of distinct regulatory mechanisms in the radiosensitivity of different types of lung cancers. Recently, miRNAs have been reported to play an important role in the radiosensitivity of cancer cells [20, 21]. In the present study, we found that miR-144-5p expression was decreased in AC and SC, but not in SCLC, compared to normal lung tissues. Moreover, the expression of miR-144-5p in AC and NSCLC cell lines was also significantly lower than in SCLC and SCLC cell lines. Intriguingly, we are the first to show that miR-144-5p was downregulated in NSCLC cells in response to IR, in a dose-dependent manner. Our findings suggest that the deregulation of miR-144-5p might contribute to the difference of radiosensitivity among various subtypes of lung cancers.

During miRNA biogenesis, only the guide strand is processed to bind to the RNA-induced silencing complex to target mRNAs, whereas the passenger strand is degraded [6, 22]. It is well documented that miR-144-3p is downregulated and serves as a tumor suppressor in a variety of tumors, including lung cancer [23], gastric cancer [24], breast cancer [25], pancreatic cancer [26], and hepatocellular carcinoma [27]. Nevertheless, the function of miR-144-5p, the guide strand from pre-miR-144, is largely unknown. It has been reported that miR-144-5p expression could be used as a prognostic biomarker for esophageal carcinoma [28], gastric cancer [29], and breast cancer [30]. In addition, Matsushita et al. [31] have reported that downregulation of the miR-144-3p and miR-144-5p cluster is frequently observed in bladder cancer cells and that miR-144-5p restoration significantly inhibits cancer cell proliferation by inducing cell cycle arrest. In agreement with these previous studies, we observed that upregulation of miR-144-5p could enhance the radiation-mediated viability inhibition, apoptosis, and growth arrest in NSCLC cells both* in vitro* and* in vivo*. Our findings suggest that miR-144-5p is not only a suitable biomarker for radiotherapeutic response, but it is a potential target in sensitizing radiotherapy in NSCLC.

To explore the mechanism underlying miR-144-5p-mediated radiosensitivity in NSCLC, we identified ATF2 as a target of miR-144-5p containing the putative miRNA response element within its 3′UTR. The AP-1 family transcription factor ATF2 is a member of the leucine-zipper domain-containing CREB/ATF transcription factor family [14]. It contributes to various cellular behaviors, from the global transcriptional activities involving cell development, proliferation, and death to the response of cells to stress signals and DNA damage [14, 32, 33]. Evidence shows deregulation of ATF2 in cancer cells, whereas complete somatic loss of ATF2 results in cell death [32, 34, 35]. ATF2 is markedly overexpressed in NSCLC [36]. Moreover, previous studies have shown that ATF2 confers cisplatin and radiation resistance of NSCLC through increasing survival and DNA repair [15, 37]. In our study, ATF2 was validated to be the direct target of miR-144-5p. miR-144-5p robustly suppressed ATF2 mRNA and protein expression in NSCLC cells. Subsequent analysis verified that miR-144-5p expression was negatively related to ATF2 abundance in lung cancer and normal lung tissues. Furthermore, overexpression of ATF2 could reverse miR-144-5p-induced cell viability inhibition, apoptosis, and growth arrest in combination with IR. In line with previous studies, our study suggests that downregulation of ATF2 is at least partially responsible for the miR-144-5p-mediated increase in radiosensitivity of NSCLC cells.

In conclusion, our study demonstrated that miR-144-5p expression was downregulated in NSCLC specimens and cell lines. IR treatment further decreased miR-144-5p expression in NSCLC cells and restoration of miR-144-5p sensitized cells to IR* in vitro* and* in vivo* via inhibition of ATF2. Our results suggest that targeting the miR-144-5p/ATF2 pathway is an effective strategy to sensitize NSCLC to radiation.
